# A High-Resolution Discrete-Time Second-Order ΣΔ ADC with Improved Tolerance to KT/C Noise Using Low Oversampling Ratio

**DOI:** 10.3390/s24175755

**Published:** 2024-09-04

**Authors:** Kyung-Chan An, Neelakantan Narasimman, Tony Tae-Hyoung Kim

**Affiliations:** 1 Centre for Integrated Circuits and Systems, Nanyang Technological University, Singapore 639798, Singapore; kyungchan.an@ntu.edu.sg; 2 Cirrus Logic, Austin, TX 78701, USA; neel.n@ieee.org

**Keywords:** oversampling ADC, high resolution, low OSR

## Abstract

This work presents a novel ΣΔ analog-to-digital converter (ADC) architecture for a high-resolution sensor interface. The concept is to reduce the effect of kT/C noise generated by the loop filter by placing the gain stage in front of the loop filter. The proposed architecture effectively reduces the kT/C noise power from the loop filter by as much as the squared gain of the added gain stage. The gain stage greatly relaxes the loop filter’s sampling capacitor requirements. The target resolution is 20 bit. The sampling frequency is 512 kHz, and the oversampling ratio (OSR) is only 256 for a target resolution. Therefore, the proposed ΔΣ ADC structure allows for high-resolution ADC design in an environment with a limited OSR. The proposed ADC designed in 65 nm CMOS technology operates at supply voltages of 1.2 V and achieves a peak signal-to-noise ratio (SNR) and Schreier Figure of Merit (FoMs) of 117.7 dB and 180.4 dB, respectively.

## 1. Introduction

Various electronic devices, such as communication equipment, biosensors, and automation devices, have become very close to our lives [1,2,3,4,5,6,7]. These electronic devices communicate the information they collect with each other and users through a network called the Internet of Things (IoT). The sensor interface is essential for IoT applications that detect and process various signals [8,9,10]. The ADC is an essential part that converts analog signals processed at the sensor interface into digital signals [11,12,13,14,15]. Slow signals, with bandwidths of less than 1 kHz, are commonly encountered in various applications, such as sensor interfaces, biomedical signal processing, and industrial instrumentation. Among these, biomedical signals, such as electrocardiography (ECG) and electroencephalography (EEG) are very small in amplitude, ranging from several microvolts to several millivolts [16,17,18,19]. These small-amplitude noise-sensitive signals require ADCs with a very high SNR. Moreover, devices powered by batteries or harvested energy, such as wearable devices, require low-power operation. SNR is a key performance metric that determines the resolution of an ADC. In the case of a low-power circuit, the signal power is limited by the low supply voltage, reducing the SNR. Therefore, reducing the noise power is the only way to increase the ADC’s resolution. Various ADCs applying the ΔΣ structure have been researched to achieve high resolution, over 15 bit [20,21,22]. The ΔΣ ADCs have oversampling and noise-shaping properties and have been studied for high-resolution applications. Oversampling reduces the main noise sources, which are quantization noise power and kT/C noise power, within the bandwidth of interest. Noise shaping reconstructs the power spectrum of quantization noise using the ΔΣ modulator with an N-th order loop filter.

**Figure 1:**
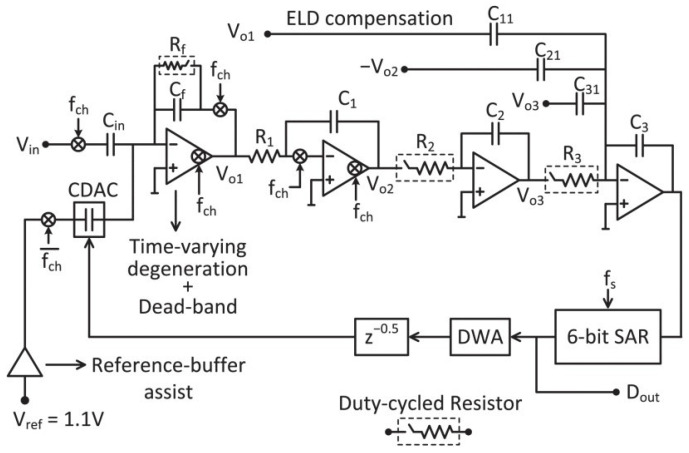
Block diagram of CT ΔΣ ADC with gain stage [20].

A continuous-time (CT) ΔΣ ADC with a gain-stage block diagram is shown in Figure 1 [20]. A conventional CT ΔΣ ADC has an active RC filter with a resistor-based digital-to-analog converter (R-DAC) as the first stage. The primary sources of noise are thermal noise from the input resistor and quantization noise. Oversampling and noise-shaping techniques reduce quantization noise, but thermal noise can only be reduced through oversampling. The use of a small resistor helps reduce thermal noise, but it results in high power consumption for the OTA and R-DAC. To address this limitation, this paper [20] proposes the use of a capacitor feedback inverting amplifier (CFIA) as a gain stage placed before the first integrator. Since the CFIA has a capacitor input, the R-DAC can be replaced with a capacitor DAC (C-DAC). Furthermore, the CFIA has a gain of four, which reduces the thermal noise power of the first integrator by 16 times. Therefore, by employing a CFIA with a gain of four, this architecture can achieve a 12 dB higher SNR compared to the conventional architecture while using the same input resistor. This ADC consists of a CFIA, a third-order loop filter, and a 6-bit quantizer. A third-order loop filter and an OSR of 40 are set to achieve a signal-to-quantization noise ratio (SQNR) of 100 dB. A 6-bit successive approximation register (SAR) ADC is used as a power-efficient multi-bit quantizer. The input resistor of the first integrator is 3 MΩ and placed after the CFIA. The thermal noise from this resistor accounts for 50% of the total in-band noise budget. This ADC achieves an SNDR of 93.5 dB with a power consumption of 4.5 μW. The gain limitation is determined by the CFIA’s linear input and output range. The gain of the CFIA is limited to four since the ADC has a 6-bit quantizer. Therefore, the ratio of thermal noise reduction is also limited to 16.

**Figure 2:**
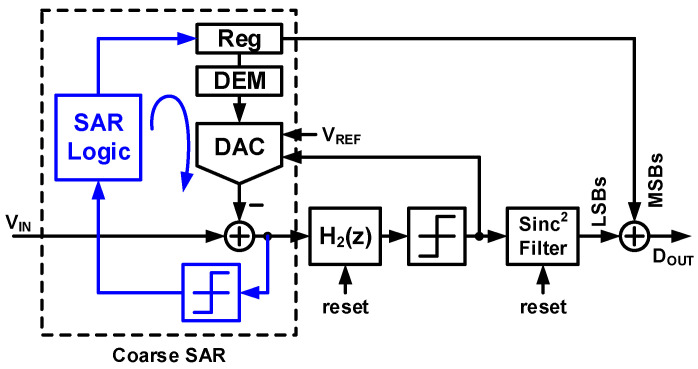
Block diagram of high-resolution incremental zoom ADC [21].

Figure 2 shows the architecture of the high-resolution incremental zoom ADC [21]. This zoom ADC operation is a two-step, coarse, and fine conversion. This ADC is a hybrid structure that combines the successive approximation register (SAR) ADC and the ΔΣ ADC. The SAR ADC is a power-efficient ADC, and the ΔΣ ADC is an oversampling and noise-shaping ADC for high resolution. The SAR ADC and ΔΣ ADC are operated for coarse and fine conversion, respectively. The coarse conversion is processed in the SAR ADC, and the output is the most significant bits (MSB) of the overall digital output. The ΔΣ ADC uses the output of the SAR ADC as the reference voltage for fine conversion. As a result, this architecture functions as a multi-bit ΔΣ ADC that uses the SAR ADC as a multi-bit quantizer. The SAR ADC has a 6-bit resolution, and the ΔΣ ADC has a 1-bit stream output. The ΔΣ ADC uses the reference voltage as the two least significant bits (LSB) with an over-ranging technique. This over-ranging mitigates the requirements of the SAR ADC. Consequently, the SAR ADC functions as a 5-bit quantizer for the ΔΣ ADC, and the overall architecture becomes a 5-bit ΔΣ ADC. The DT ΔΣ ADC has two primary noise sources, namely the quantization noise and the KT/C noise. First, to achieve an SQNR of over 120 dB, this architecture requires an OSR of 256 with a second-order loop filter and a 5-bit quantizer. The KT/C noise is not shaped by the loop filter, while the loop filter shapes the quantization noise. Thus, to obtain an SNR of 120 dB, a higher OSR than the OSR for the target SQNR is necessary. The proposed ADC in this paper employs an OSR of 2000 and a sampling capacitor of 10.2 pF to achieve a high SNR above 120 dB. The incremental-zoom ADC is designed with a 6-bit SAR ADC and a 1-bit ΔΣ ADC that have a second-order loop filter, a sampling capacitor of 10.2 pF, and an OSR of 2000. This ADC achieves a 20-bit resolution with a power consumption of 6.3 μW. However, the sequential operation of the coarse and fine conversions makes the ADC slow and limits the bandwidth to DC.

**Figure 3:**
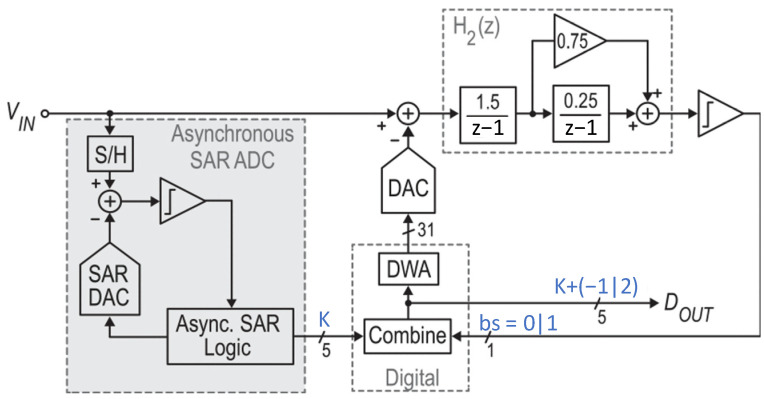
Block diagram of dynamic zoom ADC [22].

Figure 3 shows the architecture of the dynamic-zoom ADC [22], which operates similarly to the incremental-zoom ADC in reference [21]. However, the input bandwidth (BW) of the proposed ADC is 1 kHz and not limited to DC. The dynamic-zoom ADC consists of a 5-bit asynchronous SAR ADC and a 1-bit ∆Σ ADC with a second-order loop filter and a sampling capacitor of 13 pF. In this dynamic-zoom ADC, the SAR ADC and ∆Σ ADC operate simultaneously, while the incremental zoom ADC operates sequentially. The SAR ADC is an asynchronous type that continuously updates the reference voltage of the ∆Σ ADC. As a result, its reference voltage can track the input signal. To maintain a 1 kHz signal with the coarse conversion result of the SAR ADC, the ∆Σ ADC uses a 2 LSB over-ranged reference voltage. This over-ranging mitigates the SAR ADC’s requirement for tracking the input signal. The 5-bit asynchronous SAR ADC is used, which works as a 3.5-bit quantizer in the ∆Σ ADC since two LSB over-ranging is adopted. This ADC achieves an SNDR of 118.1 dB with a power consumption of 280 μW. For an SQNR of 120 dB, this architecture requires an oversampling ratio (OSR) of over 256 with a second-order loop filter. However, the proposed zoom ADC in reference [22] uses a large OSR of 1000 and a sampling capacitor of 13 pF due to the same issue described in reference [21].

In oversampling and noise-shaping ADCs, kT/C noise is not affected by noise shaping. As the target ADC resolution increases, kT/C noise becomes the primary noise source of the ADC. Increasing the capacitance to reduce this noise is one option, but large capacitances are not area efficient. Therefore, a higher oversampling ratio (OSR) is usually preferred [21,22,23,24,25,26]. However, there is a disadvantage when a given OSR is limited in system design. High-resolution ADC design is limited due to the constraint of kT/C noise [27,28,29,30,31,32,33]. Consequently, there is a clear need for research on a high-resolution ADC structure that is less affected by kT/C noise.

## 2. Proposed DT Second-Order ∆Σ ADC

### 2.1. Concept of Proposed Architecture

**Figure 4:**
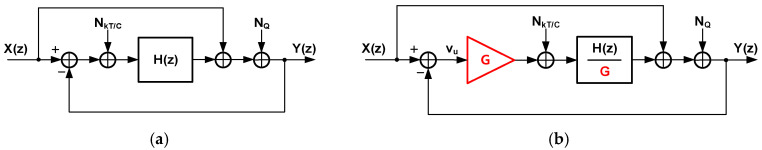
Block diagram of (**a**) conventional input feedforward ∆Σ ADC and (**b**) ∆Σ ADC employing gain-stage.

(a) For the basic concept, Figure 4a shows the conventional input-feedforward discrete-time (DT) ∆Σ ADC architecture referenced in [34,35]. H(z) represents the loop filter. N_Q_ is the quantization noise, and N_kT/C_ is the kT/C noise from the SC circuit. This ∆Σ ADC has the transfer functions described in Equations (1) and (2). In Equation (2), the STF is the signal-transfer function, NTF_kT/C_ is the noise-transfer function for kT/C noise, and NTF_Q_ is the noise-transfer function for quantization noise. As shown in Equation (2), N_kT/C_ appears equally in the output, since NTF_kT/C_ is generally almost equal to ‘1’.

Y(z) = X(z) + H(z)/(1 + H(z)) ∙ N_KT/C_ + 1/(1 + H(z)) ∙ N_Q_ (1)

STF = 1, NTF_KT/C_ = H(z)/(1 + H(z)), NTF_Q_ = 1/(1 + H(z)) (2)

Figure 4b depicts the ∆Σ ADC employing a gain stage. The gain stage is located before the loop filter. The loop-filter transfer function is H(z)/G to maintain the overall transfer function. The transfer function of an ∆Σ ADC employing the gain stage is expressed as Equations (3) and (4). Gain reduces the N_kT/C_ in Equation (3). The G^2^ will reduce the kT/C noise power.

Y(z) = X(z) + H(z)/(1 + H(z)) ∙ N_KT/C_/G + 1/(1 + H(z)) ∙ N_Q_ (3)

STF = 1, NTF_KT/C_ = H(z)/(1 + H(z)) ∙ 1/G, NTF_Q_ = 1/(1 + H(z)) (4)

**Figure 5:**
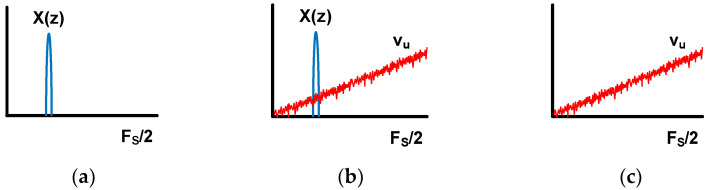
Signal spectrums in Figure 4b; (**a**) ADC input, (**b**) ADC output, and (**c**) gain-stage input.

**Figure 6:**
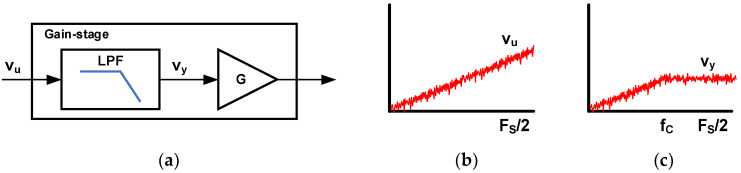
Filtered gain-stage block diagram and signal spectrum; (**a**) filtered gain-stage (**b**) spectrum before LPF (**c**) spectrum after LPF.

(b) For the filtered gain stage, since the input of the gain stage is quantization noise, an input magnitude depends on the number of bits of the quantizer. Table 1 shows the relationship between quantizer bits and V_LSB_ at a 1.2 V supply voltage. The V_LSB_ of the 5-bit quantizer is 39 mV. In this case, if the linear output range of the gain stage is 200 mV, the gain is limited to five. The filtered gain stage is proposed to overcome this limitation. Figure 5 shows the signal spectrums of each point in Figure 4b. Figure 5a,b illustrates the input X and output Y spectrums, respectively. Figure 5c is the gain-stage input spectrum, which is the noise-shaped quantization error. As shown in Figure 5c, the maximum magnitude of quantization noise is from the high-frequency term, limiting the maximum gain of the gain stage. The gain stage with first-order LPF is shown in Figure 6a. The LPF is placed before the gain stage to reduce the high-frequency components of the quantization noise. Adding LPF provides two advantages. First, as shown in Figure 6b,c, the LPF reduces the maximum amplitude of shaped quantization noise and helps the gain stage have a large gain. The reduction ratio is equivalent to the ratio of the loop filter unity–gain frequency, F_T,H(z)_, and the cut-off frequency, f_C_, of the filter. The unity-gain frequency of the loop filter is written as Equation (5), and the reduction ratio is F_S_/(2π∙f_C_). F_S_ is the sampling frequency. The second advantage is avoiding noise aliasing. In the DT ∆Σ ADC, the sampling by the SC circuit aliases the noises outside the F_S_/2 [36]. The LPF prevents noise-aliasing since the LPF filters the white noise of the gain stage from f_C_.

F_T,H(z)_ = F_S_/2π (5)

### 2.2. Proposed Second-Order ∆Σ ADC Architecture

**Figure 7:**
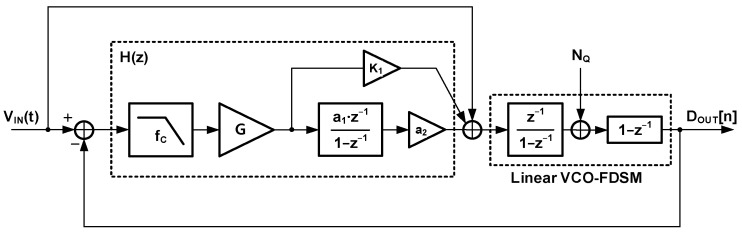
Proposed second-order ∆Σ ADC architecture.

Figure 7 shows the proposed second-order ∆Σ ADC based on an input feedforward architecture. The input feedforward architecture has the advantage that an input of the loop filter is only the quantization noise. This architecture also reduces the output swing range of the first integrator and mitigates the design requirements of the integrator. The proposed ∆Σ ADC adopts the new loop filter consisting of the filtered gain stage and the DT loop filter. The filtered gain stage is a combination of the gain stage and the LPF. The LPF reduces the gain-stage input amplitude, which is the quantization noise. The filtered signal helps the gain stage have a higher gain. Also, a multi-bit quantizer is used for a smaller filtered gain-stage input. This ADC targets general sensor applications with a signal bandwidth of 1 kHz, such as smart sensors, biomedical imaging, and portable instrumentation. The sampling frequency is set to 512 kHz by the given OSR and BW. By selecting the 5 kHz cutoff frequency for the LPF, the filtered gain stage can have a gain of 50. It is expected that the gain reduces the kT/C noise power 2500 times.

**Figure 8:**
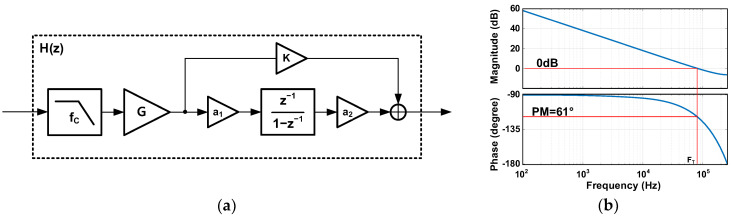
Proposed ADC’s (**a**) loop-filter block diagram and (**b**) frequency response.

(a) For consideration of the proposed loop filter, the LPF cutoff frequency is 5 kHz to reduce the quantization noise at high frequencies. The amount of noise reduction by F_S_/(2π∙f_C_) is about 16 times, with a cutoff frequency of 5 kHz and a sampling frequency of 512 kHz. From Table 1, the V_LSB_ of the 5-bit quantizer is 39 mV. For a 5-bit quantizer and a 1.2 V supply voltage, the signal amplitude after LPF is 2.4375 mV. Thus, the maximum available gain is 82, assuming the linear output range of the gain stage is 200 mV. In a first-order proposed loop filter, the gain is set to 50.

(b) For the stability of the proposed loop filter, in the loop filter with a filtered gain stage, the LPF of the filtered gain stage introduces another pole in the transfer function. This stability issue requires frequency compensation, and a feedforward path can be adopted to address this issue. The feedforward path provides a zero and restores the unity-gain frequency F_T_ to an original loop filter of F_S_/2π. Figure 8a depicts the block diagram of the proposed ADC’s loop filter with a feedforward path. The open-loop transfer function of the proposed ADC is represented as H_ADC_(z) = H(z)∙z^−1^, where z^−1^ denotes the delay in the linear VCO-FDSM. H(z) includes the gain stage, SC integrator, and feedforward path with K_1_. The s-domain block can be converted to z-domain using the Laplace transform in Equation (6), and α is a coefficient used to correct the DC gain. The α can be determined by evaluating H_1_(1) = 1.

(6) $$H_{1}\left(s\right)=\frac{1}{s+a}↔H_{1}\left(z\right)=\alpha \cdot \frac{z}{z-\;e^{-\mathrm{aTs}}}$$

Then, H(z) is expressed as Equation (7), where ω_C_ is 2·π·f_C_ and T_S_ is 1/F_S_. The parameters f_C_, G, a_1_, a_2_, and K_1_ are set to the same values as those in Table 2. Figure 8b shows the frequency response of the transfer function of the proposed ADC’s loop filter in Figure 8a. The frequency response has a unity-gain frequency F_T_ restored by the feedforward path with K_1_, and the F_T_ is F_S_/2π. The open-loop transfer function has a phase margin of 61°, which means that the proposed ADC is still stable.

(7) $$H(z)=G\times \frac{1-e^{-\omega_{C}T_{S}}}{1-e^{-\omega_{C}T_{S}}\cdot z^{-1}}\times \left(a_{1}\cdot a_{2}\cdot \frac{z^{-1}}{1\;-z^{-1}}+K_{1}\right)$$

### 2.3. Behavioral Simulation

**Figure 9:**
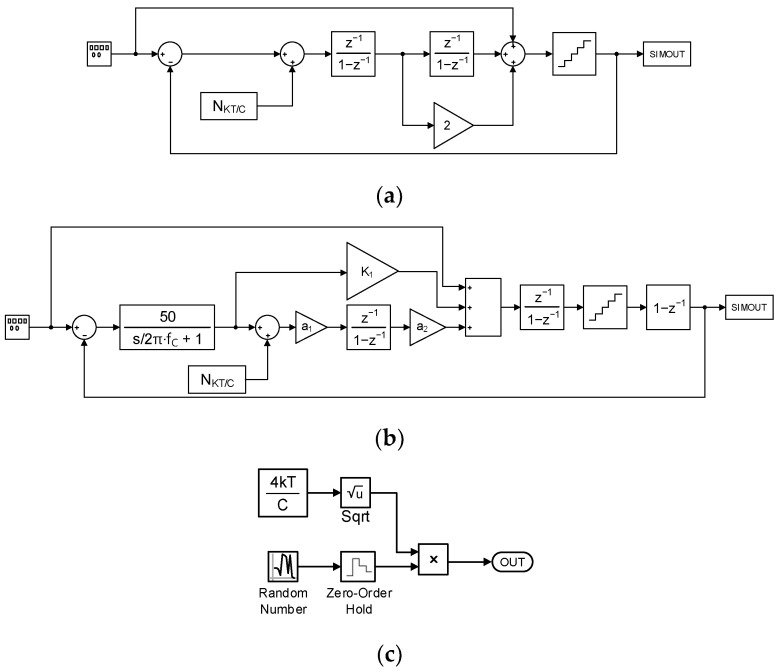
Behavioral simulation model. (**a**) Conventional ∆Σ ADC, (**b**) proposed ∆Σ ADC, and (**c**) kT/C noise.

For comparison, both the conventional ∆Σ ADC and the proposed ∆Σ ADC are modeled and simulated with MATLAB Simulink. Figure 9 shows the conventional second-order ∆Σ ADC model in (a), the proposed second-order ∆Σ ADC model in (b), and the kT/C noise model in (c). The kT/C noise block is placed before the SC integrator block, since the switched-capacitor circuit causes the kT/C noise in the SC integrator. The maximum kT/C noise of the SC integrator is 2kT/C_S_, where the C_S_ is the sampling capacitance [37]. The noise coefficient is set to 4 kT/C_S_, assuming the differential circuit. The OSR is set to 256. The C_S_ of 150 fF is chosen, which is the C_S_ requirement for a 15-bit resolution of the conventional ∆Σ ADC at a supply voltage of 1.2 V. For the proposed ∆Σ ADC, the kT/C noise power is reduced 2500 times by the gain of the filtered gain stage. The reduction in noise power implies an increase in SNDR, calculated using the equation SNDR = 10·log_10_(P_sig_/P_noise_). Consequently, a 2500-times decrease in noise power is equivalent to an approximately 33 dB increase in SNDR by 10·log_10_(2500), corresponding to an increase of about 5.2 bits in the ENOB. Therefore, a 20-bit resolution is expected in the proposed ∆Σ ADC.

**Figure 10:**
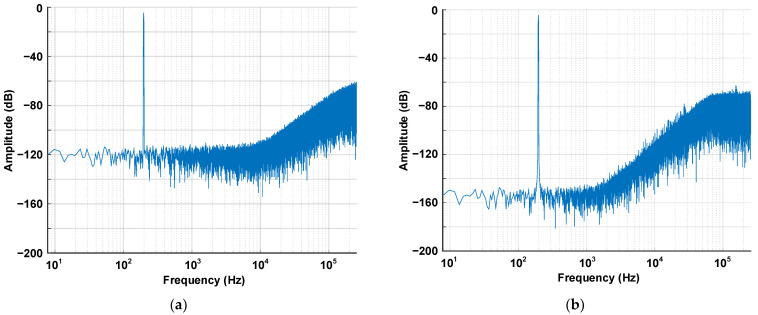
Simulated 2^18^-point output PSD of (**a**) conventional ∆Σ ADC, and (**b**) proposed ∆Σ ADC.

Figure 10 shows the simulation results for Figure 9. Figure 10a,b shows the output spectrum of the conventional ∆Σ ADC and the proposed ∆Σ ADC, respectively. The proposed ∆Σ ADC achieves an SNDR and ENOB of 122.7 dB and 20.1 bits, while the conventional ∆Σ ADC has an SNDR and ENOB of 89.1 dB and 14.5 bits. As expected, the proposed ∆Σ ADC has a 5-bit higher ENOB than the conventional ∆Σ ADC.

## 3. Circuit Implementation and Noise Contribution

**Figure 11:**
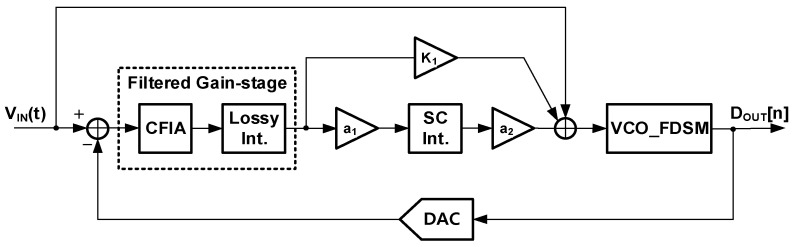
Block diagram of proposed 2nd-order ∆Σ ADC.

Figure 11 shows the circuit diagram of the second-order ∆Σ ADC employing the proposed loop filter, which consists of the filtered gain stage, SC integrator, compensation paths, and linear VCO-FDSM as a quantizer. The target ENOB and BW are 12-bit and 1 kHz, respectively, and the OSR is 256. The supply voltage, the sampling frequency, and the filter cutoff frequency are 1.2 V, 512 kHz, and 5 kHz, respectively. The main idea behind the proposed loop filter is to add the filtered gain stage in front of the DT loop filter to reduce the effects on the kT/C. This concept enables the design of high-resolution DT ∆Σ ADCs at low OSR. The total gain of the filtered gain stage is set to 50. The filtered gain stage consists of the chopper, the capacitor feedback inverting amplifier (CFIA), and the active lossy integrator. The filtered gain stage performs the delta (Δ) function with C-DAC, since the input component of the chopper CFIA is the capacitor. The CFIA has a gain of five, which depends on the quantizer. The gain of the CFIA mitigates the design requirements of the following blocks. The active lossy integrator has a DC gain and a low-pass filtering function. The filtering reduces the magnitude of the feedback signal and allows for a larger DC gain. The loop filter consists of the active SC integrator. The coefficient a_1_ is the sampling and integration capacitors ratio, C_S_/C_INT_. Linear VCO-FDSM [36] is adopted as a 5-bit quantizer, including the three-input adder. The coefficients a_2_ and K_1_ are implemented by weighting the three input capacitances of the quantizer. Noise power can be represented by P_noise_ = S_noise_ × BW in the frequency domain. The S_noise_ is the noise power spectral density (PSD), and BW is the bandwidth. The required S_noise_ calculated is 4.38 × 10^−16^ V^2^/Hz, since the BW is 1 kHz in this research. The noise PSD summation of the proposed ADC must be less than the target noise budget S_noise_. 

### 3.1. Filtered Gain Stage

**Figure 12:**
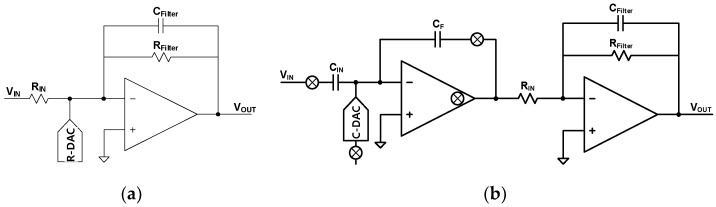
Circuit diagram of (**a**) conventional active lossy integrator, and (**b**) proposed filtered gain-stage.

An active lossy integrator can easily implement a filtered gain stage, as shown in Figure 12a. The lossy integrator utilizes an R-DAC for the delta function. This structure functions as a filtered gain stage. The DC gain and cutoff frequency are defined by Equations (8) and (9).

A_Filter_ = R_Filter_/R_IN_ (8)

(9) $$f_{C}=1/(2\pi \;\cdot \;R_{\mathrm{Filter}}\;\cdot \;C_{\mathrm{Filter}})$$

However, there are noise and power-efficiency issues. The input resistor produces a thermal noise of 4kTR_IN_. The OTA also has thermal and flicker noise that appears directly on the output. A smaller input resistance reduces thermal noise, but the OTA and R-DAC require more power. Thus, this structure is not suitable for a power-efficient system. The proposed solution is the filtered gain stage, which consists of the CFIA and an active lossy integrator, as shown in Figure 12b. The cutoff frequency f_C_ is 5 kHz. The total gain is 50. The gains of the CFIA and the lossy integrator are 5 and 10, respectively. The CFIA has been used for low-noise applications in [20]. The gain of CFIA mitigates the design requirements of the active lossy integrator. Additionally, the capacitor input allows for the use of C-DAC instead of R-DAC and eliminates the thermal noise of the R-DAC. Finally, the proposed filtered gain stage reduces the amplitude of high-frequency quantization noise above 5 kHz and provides a high gain of 50 to mitigate the kT/C noise of the following SC integrator.

### 3.2. Capacitor Feedback Inverting Amplifier

**Figure 13:**
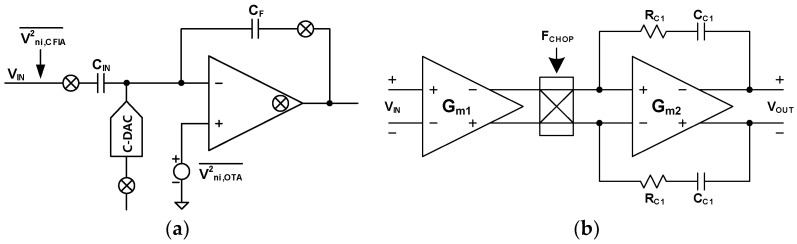
Circuit diagram of (**a**) chopping CFIA with C-DAC and (**b**) OTA for chopping CFIA.

Figure 13a shows the chopping CFIA block diagram with the noise model. The C_IN_ and C_F_ are the input and feedback capacitors, respectively. The CFIA has the input capacitor, while the conventional inverting amplifier has the input resistor. Since the input component is a capacitor, the DAC type is C-DAC. This structure eliminates the thermal noise caused by the resistor. The gain of the CFIA is derived from Equation (10).

A_CFIA_ = C_IN_/C_F_ (10)

The CFIA’s input capacitor blocks a DC signal. The CFIA can process the DC signal by applying the chopping technique with the CFIA. The chopping technique also has the advantage of shifting the flicker noise of the OTA to the chopping frequency. As a result, only the thermal noise of the OTA is present in the bandwidth of interest [38]. The CFIA is primarily affected by thermal noise of the OTA, as shown in Figure 13a. The flicker noise of the OTA can be disregarded, since it will be shifted to the chopping frequency. 

The input-referred noise of the CFIA, $\bar{{V^{2}}_{\mathrm{ni},\mathrm{CFIA}}}$, is larger than $\bar{{V^{2}}_{\mathrm{ni},\mathrm{OTA}}}$. The relationship is demonstrated in Equation (11), where A_CFIA_ represents the gain of the CFIA [39]. When A_CFIA_ is 5, the $\bar{{V^{2}}_{\mathrm{ni},\mathrm{CFIA}}}$ is 1.44 times larger than $\bar{{V^{2}}_{\mathrm{ni},\mathrm{OTA}}}$.

(11) $$\bar{V_{\mathrm{ni},\mathrm{CFIA}}^{2}}=\frac{{\left(A_{\mathrm{CFIA}}+1\right)}^{2}}{A_{\mathrm{CFIA}}^{2}}\bar{V_{\mathrm{ni},\mathrm{OTA}}^{2}}$$

**Figure 14:**
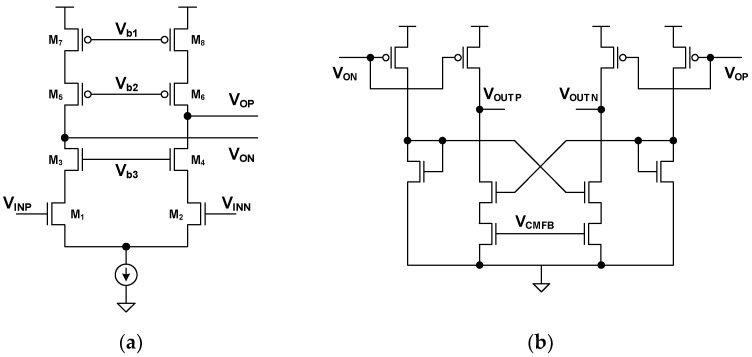
OTA circuit detail of (**a**) first stage G_m1_ and (**b**) second stage G_m2_.

The OTA block diagram for the CFIA is shown in Figure 13b. The OTA consists of a two-stage structure. The first stage is a telescopic amplifier, and the second stage is an output buffer based on a common-source (CS) amplifier. Figure 14a,b shows the circuit details of the first and second stages, respectively. The transistor-level analysis of this is detailed in reference [40]. The first stage gain reduces the input-referred noise of the second stage. As a result, the noise from the first stage is dominant. The single MOSFET noise is $\bar{V_{n,\mathrm{MOSFET}}^{2}}=4\;\mathrm{kT}\gamma /g_{m}$. The k is Boltzmann’s constant, and T is the absolute temperature. The γ is the excess noise coefficient, and g_m_ is the transconductance. The value of γ is two-thirds for the strong inversion region and one-half for the weak inversion region. The input-referred noise of the telescopic amplifier is defined by Equation (12).

(12) $$\bar{V_{\mathrm{ni},\mathrm{OTA}}^{2}}\approx 4\mathrm{kT}\left(2\frac{\gamma_{1,2}}{g_{m1,2}}+2\frac{\gamma_{7,8}\cdot g_{m7,8}}{g_{m1,2}^{2}}\right)$$

Since chopping is applied, the flicker noise is negligible. The noise of the M_3–6_ is negligible because its contribution is small in the low-frequency region. According to Equations (11) and (12), a g_m1,2_ value of 300 μS makes a $\bar{{V^{2}}_{\mathrm{ni},\mathrm{CFIA}}}$ of 1.325 × 10^−16^ V^2^/Hz, which accounts for 30% of the noise budget S_noise_, assuming that M_1_ and M_2_ are biased in the weak inversion region and the g_m1,2_ is two-times larger than g_m7,8_.

**Figure 15:**
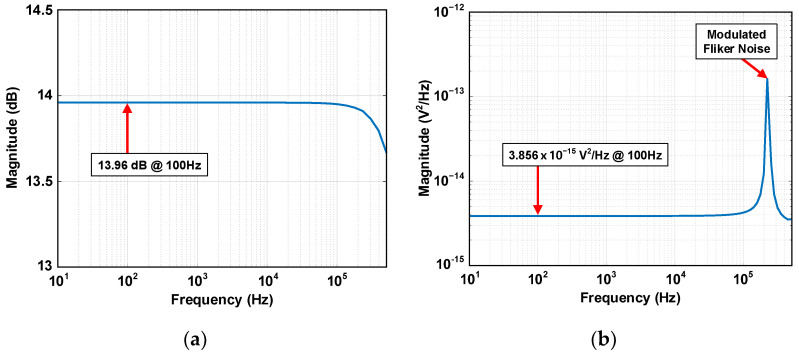
Transistor-level simulation results of chopping CFIA’s (**a**) frequency response and (**b**) output noise power density.

A transistor-level simulation is performed. The input capacitor C_IN_ and feedback capacitor C_F_ of the chopping CFIA are 500 fF and 2.5 pF, respectively. The chopping frequency F_CHOP_ is 256 kHz. The gain of the CFIA is determined by the ratio of C_IN_ and C_F_, which is 5 V/V or 13.97 dB on the dB scale. Figure 15a shows the frequency response of the chopping CFIA, which has a flat gain of 13.96 dB within the target bandwidth. In CFIA, g_m1_ of the first stage is set to 300 μS, and the input-referred noise hand-calculation result is 1.325 × 10^−16^ V^2^/Hz. This value accounts for 30% of the total noise budget. Figure 15b shows the chopping CFIA’s output noise power density $\bar{{V^{2}}_{\mathrm{no},\mathrm{CFIA}}}$. The chopping CFIA has $\bar{{V^{2}}_{\mathrm{no},\mathrm{CFIA}}}$ of 3.856 × 10^−15^ V^2^/Hz within the target bandwidth, and the flicker noise of the OTA is up-modulated with F_CHOP_. Since the gain of the CFIA is 5 V/V, the $\bar{{V^{2}}_{\mathrm{ni},\mathrm{CFIA}}}$ is calculated to 1.542 × 10^−16^ V^2^/Hz, which accounts for 35.2% of the total noise budget. The noise error between the hand calculation and the simulation comes from the chopping switch’s thermal noise and other OTA components’ noise.

### 3.3. Active Lossy Integrator

**Figure 16:**
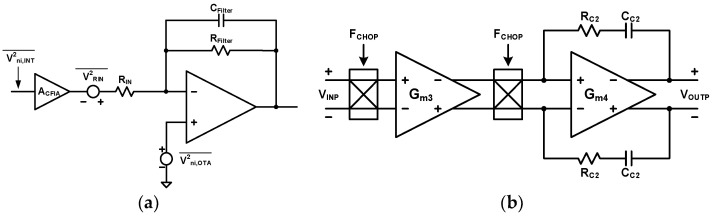
Circuit diagram of (**a**) active lossy integrator with gain of CFIA, and (**b**) OTA for active lossy integrator.

Since the CFIA has a gain limitation, the active lossy integrator in Figure 16a can be used to add extra gains [41]. This LPF also has thermal noise generated by an input resistor, but the CFIA gain reduces this noise. Larger resistors can be used for the same amount of noise power. This circuit’s gain and cutoff frequency are as specified in Equations (8) and (9), respectively.

The active lossy integrator has two main noise sources, as shown in Figure 16a, namely the thermal noises of resistor $\bar{{V^{2}}_{\mathrm{RIN}}}$ and OTA $\bar{{V^{2}}_{\mathrm{ni},\mathrm{OTA}}}$. The OTA noise equation is the same as Equation (12). The input-referred noise of the active lossy integrator V^2^_niFilter_ is derived by Equation (13).

(13) $$\bar{V_{\mathrm{ni},\mathrm{Filter}}^{2}}=\frac{1}{A_{\mathrm{CFIA}}^{2}}\frac{{\left(A_{\mathrm{Filter}}+1\right)}^{2}}{A_{\mathrm{Filter}}^{2}}\bar{V_{\mathrm{ni},\mathrm{OTA}}^{2}}$$

A_CFIA_ and A_Filter_ are the gains of the CFIA and active lossy integrator. Since this block is located after the CFIA with a gain of A_CFIA_, the $\bar{{V^{2}}_{\mathrm{ni},\mathrm{Filter}}}$ is reduced by the A^2^_CFIA_. Figure 16b illustrates the OTA circuit diagram of the active lossy integrator. The OTA consists of two Gm stages, two chopping switches, and Miller compensation. The chopping switches are adopted to remove the flicker noise effect. The G_m3_ and G_m4_ structures are the same as the G_m1_ and G_m2_ of Figure 14, respectively. When the A_CFIA_ is 5 and the A_Filter_ is 10, the $\bar{{V^{2}}_{\mathrm{ni},\mathrm{Filter}}}$ is 0.041 ×$\;\bar{{V^{2}}_{\mathrm{ni},\mathrm{OTA}}}$. A g_m1,2_ of 30 μS makes a $\bar{{V^{2}}_{\mathrm{ni},\mathrm{Filter}}}$ of 4.45 × 10^−17^ V^2^/Hz, which is 10% of the noise budget S_noise_. 

The second noise source is the input resistor’s thermal noise, $\bar{{V^{2}}_{\mathrm{RIN}}}$, which is expressed as $\bar{V_{\mathrm{RIN}}^{2}}=4\mathrm{kT}R_{\mathrm{IN}}$. The input-referred resistor thermal noise $\bar{{V^{2}}_{\mathrm{ni},\mathrm{RIN}}}$ is represented by Equation (14). The CFIA also reduces the $\bar{{V^{2}}_{\mathrm{RIN}}}$ by A^2^_CFIA_. A R_IN_ of 100 kΩ makes a $\bar{{V^{2}}_{\mathrm{ni},\mathrm{RIN}}}$ of 1.325 × 10^−16^ V^2^/Hz, which is 30% of the noise budget S_noise_. The total input-referred noise generated by the active lossy integrator is $\bar{V_{\mathrm{ni},\mathrm{INT}}^{2}}=\bar{V_{\mathrm{ni},\mathrm{Filter}}^{2}}+\bar{V_{\mathrm{ni},\mathrm{RIN}}^{2}}$.

(14) $$\bar{V_{\mathrm{ni},\mathrm{RIN}}^{2}}=4\mathrm{kT}R_{\mathrm{IN}}/A_{\mathrm{CFIA}}^{2}$$

**Figure 17:**
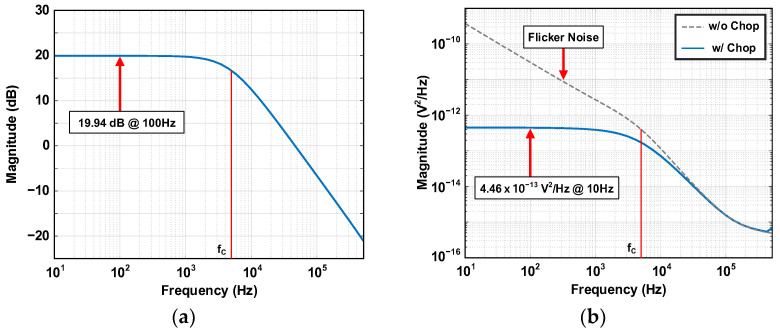
Chopping lossy integrator’s (**a**) frequency response and (**b**) output noise power density with and without chopping.

The input resistor R_IN_ of the lossy integrator is 100 kΩ, and the feedback resistor R_Filter_ and capacitor C_Filter_ for a cutoff frequency of 5 kHz are 1 MΩ and 32 pF, respectively. The chopping frequency F_CHOP_ is 256 kHz. The DC gain of the lossy integrator is determined by the ratio of R_IN_ and R_Filter_, which is 10 V/V and 20 dB on the dB scale. Figure 17a shows the frequency response of the chopping lossy integrator, which has a flat gain of 19.94 dB within the target bandwidth of 1 kHz. The lossy integrator has a cutoff frequency f_C_ of 5 kHz and a −20 dB/Dec slope above f_C_. In the lossy integrator, g_m3_ of the first stage is set to 30 μS, and the hand-calculated input-referred noise result at the CFIA input is 4.45 × 10^−17^ V^2^/Hz. This value accounts for 10% of the total noise budget. For the thermal noise of an R_IN_ of 100 kΩ, the hand-calculated input-referred noise at the CFIA input is 1.325 × 10^−16^ V^2^/Hz. This value accounts for 30% of the total noise budget. Therefore, $\bar{{V^{2}}_{\mathrm{ni},\mathrm{INT}}}$, the hand-calculated input-referred noise of the lossy integrator at the CIFA’s input node, is 1.77 × 10^−16^ V^2^/Hz, which accounts for 40.4% of the total noise budget. Figure 17b shows the lossy integrator’s output noise power densities $\bar{{V^{2}}_{\mathrm{no},\mathrm{INT}}}$ with and without chopping. Without chopping, the lossy integrator suffers from a large low-frequency noise due to the flicker noise of OTA. Chopping modulates this flicker noise and moves it into out of band. The lossy integrator with chopping has a ${V^{2}}_{\mathrm{no},\mathrm{INT}}$ of 4.46 × 10^−13^ V^2^/Hz within the target bandwidth. Since the total gain of the gain stage is 50 V/V, the $\bar{{V^{2}}_{\mathrm{no},\mathrm{INT}}}$ is calculated to 1.784 × 10^−16^ V^2^/Hz, which accounts for 40.74% of the total noise budget.

### 3.4. Switched-Capacitor Integrator

**Figure 18:**
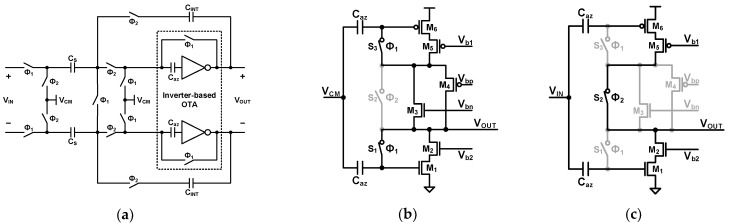
Circuit diagram of (**a**) switched-capacitor integrator and inverter-based OTA’s (**b**) sampling phase Φ_1_ and (**c**) integration phase Φ_2_.

The first-order loop filter consists of a switched-capacitor (SC) integrator, as shown in Figure 18a. The SC integrator works with the two-phase clock of Φ_1_ and Φ_2_. Φ_1_ is the sampling phase, and Φ_2_ is the integration phase. This SC integrator structure is parasitic insensitive [40], which has a transfer function given by Equation (15).

(15) $$H_{\mathrm{int}1}\left(z\right)=a_{1}\cdot \frac{z^{-1/2}}{1\;-z^{-1}}$$

Since the SC integrator produces the output at Φ_2_, not Φ_1_, it has a half delay z^−1/2^. The coefficient a_1_ is the ratio of C_S_ and C_INT_, C_S_/C_INT_. An inverter-based OTA is a desirable solution for a power-efficient circuit, and Figure 18b,c shows the circuit detail. The transistor-level analysis of this is detailed in reference [31]. The cascode inverter architecture provides a high DC gain. The biasing floating current source and auto-zeroing capacitor C_az_ are adopted for setting the operating point. In the sampling phase Φ_1_ in Figure 18b, the floating current source provides a bias current, and S_1_ and S_3_ connect the input and output nodes of the inverter. The shorted inverter makes the switching point according to the bias current. The DC offset caused by the voltage difference of V_CM_ and inverter switching point is charged in the C_az_. In the integration phase Φ_2_ in Figure 18c, S_2_ turns off the floating current source, and S_1_ and S_3_ disconnect the input and output nodes. The C_az_ compensates the inverter DC offset and sets the operating point to V_CM_. 

**Figure 19:**
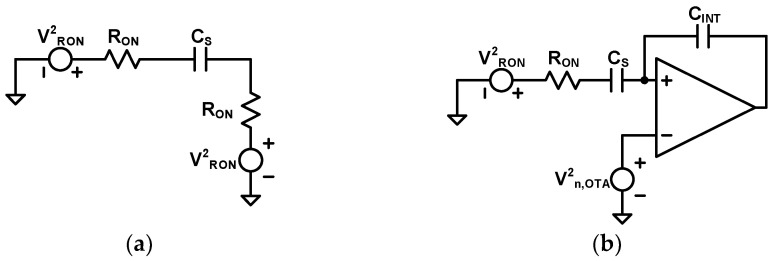
Noise analysis of SC integrator (**a**) sampling phase (**b**) integration phase.

The SC integrator operation for noise analysis is represented in Figure 19 [37]. As shown in Figure 19a, the sampling phase has the noise power P_n,CINΦ1_ of the input SC circuit, which is kT/C_S_. The integration phase in Figure 19b produces the two noise powers, P_n,CINΦ2_ and P_n,OTA_, from the input SC circuit and OTA. Assuming that OTA has the noise V^2^_n,OTA_ as given by Equation (16), P_n,CINΦ2_ and P_n,OTA_ are represented by Equations (17) and (18), respectively.

(16) $$V_{n,\mathrm{OTA}}^{2}=\frac{16\mathrm{kT}}{{3g}_{m}}n_{f}$$

(17) $$P_{n,\mathrm{CIN}\Phi 2}=\frac{\mathrm{kT}}{C_{S}}\times \frac{x}{1+x}$$

(18) $$P_{n,\mathrm{OTA}}=\frac{4\mathrm{kT}}{3C_{S}}\times \frac{n_{f}}{1+x}$$

The noise coefficient n_f_ depends on the OTA architecture and is 1.25~1.5 in this work. The x is 2R_ON_g_m_, where R_ON_ and g_m_ are the switch resistance and OTA transconductance. The total noise power of the SC integrator is the sum of kT/C_S_, Equations (17) and (18). In the differential architecture, only P_n,CINΦ1_ and P_n,CINΦ2_ become double. Therefore, the total SC integrator noise power P_n,SCINT_ for the differential architecture is Equation (19).

(19) $$P_{n,\mathrm{SCINT}}=\frac{\mathrm{kT}}{C_{S}}\times \left(\frac{4n_{f}/3+2+4x}{1+x}\right)$$

When the x has an infinite value, the P_n,SCINT_ has a maximum noise power of 4 kT/C_S_. The input-referred noise density V^2^_ni,SCINT_ is represented in Equation (20), where F_S_ is the sampling frequency.

(20) $$V_{\mathrm{ni},\mathrm{SCINT}}^{2}=\frac{1}{G^{2}}\times \frac{4\mathrm{kT}}{C_{S}F_{S}}$$

G is the gain of the filtered gain stage, which reduces the SC integrator noise by G^2^. When the G is 50, the V^2^_ni,SCINT_ is reduced 2500 times. A C_S_ of 300 fF makes V^2^_ni,SCINT_ of 4.31 × 10^−17^ V^2^/Hz, which is 10% of the noise budget S_noise_.

### 3.5. Linear VCO-FDSM as 5-Bit Quantizer with Adder

**Figure 20:**
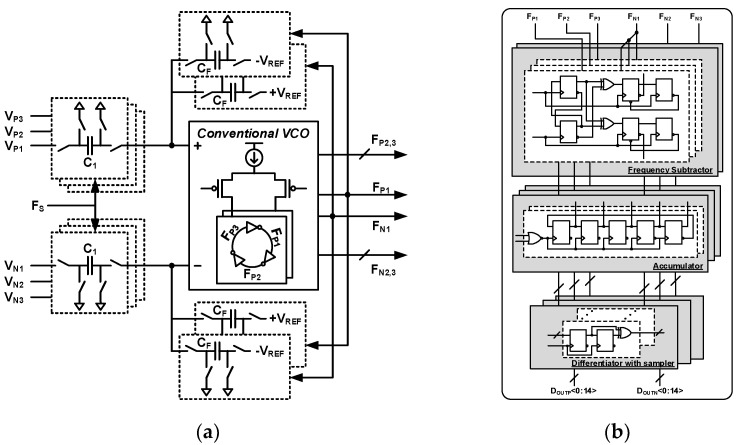
Circuit diagram of (**a**) linear VCO with 3-input adder and (**b**) fully digital FDSM with 30-bit thermometer output code.

Linear VCO-FDSM [36], which consists of a linear VCO and a fully digital frequency delta–sigma modulator (FDSM), is adopted as a quantizer having a first-order noise-shaping property. Figure 20a shows a detailed circuit diagram of the linear VCO with an adder. As a three-input adder, the linear VCO has the three-input SC, C_1_, C_2_, and C_3_. The adder has three inputs, namely input feedforward, filtered gain-stage output, and SC integrator output. The coefficient values a_1_ and K_1,_ required for each input shown in Figure 11, are implemented by weighting the input capacitor ratio C_1_:C_2_:C_3_. The conventional VCO is based on a three-stage ring oscillator. The linear VCO-FDSM uses all three inverter outputs of the ring oscillator to realize a 5-bit quantizer. The outputs of the differential linear VCO are F_P1–3_ and F_N1–3_. The maximum output frequency of the linear VCO-FDSM is five times the sampling frequency F_S_.

Figure 20b shows the fully digital FDSM circuit diagram, consisting of the frequency subtractor, accumulator, and differentiator with the sampler. The output of the linear VCO becomes the input of the frequency subtractor. Three frequency subtractors are a group. One group compares F_P1–3_ and F_N1_, and the other group compares F_N1–3_ and F_P1_ to the output the frequency difference. The accumulator is based on the shift register. There are three groups, and each group has two accumulators. To handle the maximum output frequency of 5F_S_ of the linear VCO, the accumulator is composed of a five-stage shift register. Each stage state is shifted one step at every input rising edge, and the number of changed states is the total accumulated value. The accumulator has a transfer function of z^−1^/1-z^−1^ with the linear VCO. This accumulated value is quantized and differentiated every F_S_ by the following differentiator with the sampler. The unit differentiator consists of two registers and an XOR gate. The first register samples the input at every F_S_. The second register makes a delay z^−1^, and an XOR gate subtracts the first and second register’s outputs. The differentiator has three groups, and each group is composed of 10-unit differentiators. The differentiator has a 30-bit thermometer code, and its transfer function is 1 − z^−1^. A 30-bit thermometer code is almost equivalent to a 5-bit binary code. The shift-register-based accumulator provides DEM characteristics to the output of the linear VCO-FDSM. A quantizer with intrinsic DEM characteristics mitigates mismatch errors in feedback DACs without additional DEM blocks.

In the second-order ΣΔ ADC, the quantizer obtains additional SQNR according to the OSR, as shown in Equation (21).

(21) $${\mathrm{SQNR}}_{\mathrm{extra}}=15.05\times \log2(\mathrm{OSR})\;-\;12.9$$

The proposed ADC has an OSR of 256, so SQNR_extra_ is 107.5 dB. And for a 5-bit quantizer, the total SQNR is 139.3 dB. This is 17.14 dB larger than the SNDR of the 20-bit ADC, 122.16 dB. In other words, the quantization noise power is more than 15 dB lower than the target noise power of the 20-bit ADC, which is less than 5% of the total noise power budget.

### 3.6. Proposed Second-Order DT ∆Σ ADC with Filtered Gain-Stage

**Figure 21:**
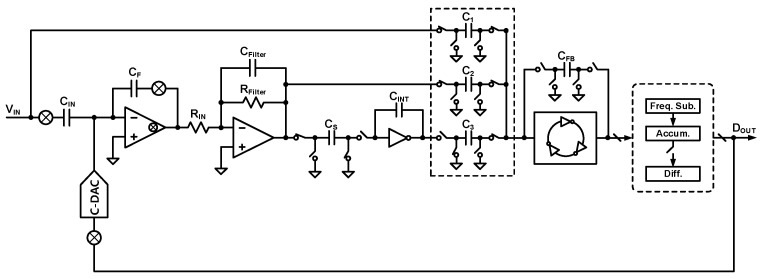
Simplified circuit diagram of proposed 2nd-order ΔΣ ADC.

Figure 21 shows the circuit diagram of the proposed second-order ΔΣ ADC. The proposed ADC is a first-order input feedforward structure. The loop filter consists of an SC integrator. The main idea is to apply a filtered gain stage to reduce the effect of kT/C input-referred noise caused by the loop filter. The filtered gain stage consists of a CFIA and an active lossy integrator to achieve low input-referred noise and high DC gain. The CFIA includes a chopping technique, which allows DC input signals and eliminates flicker noise and DC offset. The active lossy integrator suppresses high-frequency signals with high amplitude and provides high DC gain. By the high gain of the filtered gain stage, the value of the input capacitor of the SC integrator to achieve the target performance is significantly relaxed. The multi-bit quantizer and three-input adder are implemented with a linear VCO-FDSM. The linear VCO has three input SC circuits for the adder function. The output of VCO-FDSM is a 30-bit thermometer code, equivalent to a 5-bit binary code. 

**Figure 22:**
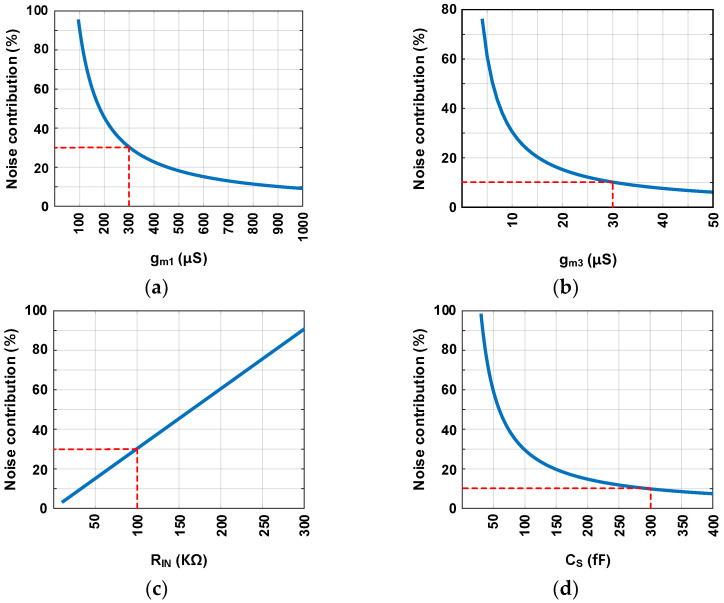
Noise contribution of (**a**) OTA in CFIA, (**b**) OTA in active lossy integrator, (**c**) R_IN_ in active lossy integrator, and (**d**) C_S_ in SC integrator.

Table 3 shows the main parameters and values used in circuit implementation, as well as the simulated noise contributions of the main blocks. The primary noise sources of the proposed ADC are the CFIA and active lossy integrator. In Section 3.2 and Section 3.3, the validity of the noise analysis method is confirmed based on the hand-calculation values and transistor-level simulation results. Figure 22 plots the noise contributions according to the design parameters of each major block based on this analysis method. The parameters affecting the noise of the block CFIA, active lossy integrator, and SC integrator are g_m1_, R_IN_, and C_S_. Figure 22a,b are the values for OTA of CFIA and active lossy integrator, respectively. Figure 22c,d are the noise contributions for the active lossy integrator’s input resistance and the SC integrator’s input capacitor. To ensure that the block CFIA, active lossy integrator, and SC integrator each account for 30%, 30%, and 10% of the noise budget, the gm1, R_IN_, and C_S_ are 300 μS, 100 kΩ, and 300 fF, respectively. 

Table 3 shows the parameters used in the main block design and the simulated noise results based on the analysis. The CFIA and active lossy integrator account for the largest portion at 35.2% and 40.74%, respectively, while the SC integrator and quantizer account for 10% and more than 5%, respectively. Based on the analysis, it can be confirmed that the sum of the noise proportions of each simulated block is 90%, which is within the target noise budget.

## 4. Simulation Results

**Figure 23:**
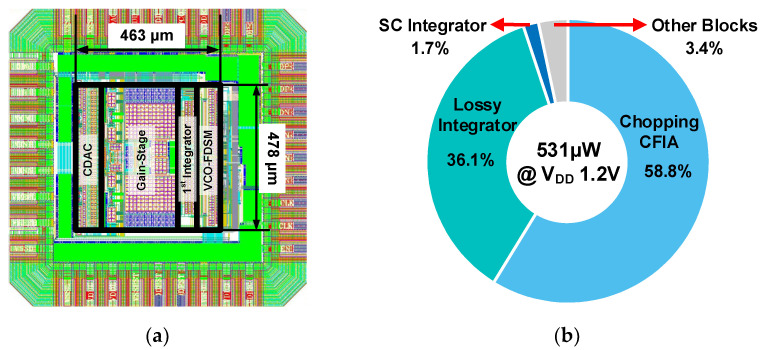
(**a**) Layout of proposed ADC and (**b**) power consumption.

Figure 23a shows the proposed second-order ΔΣ ADC chip layout. The proposed 2nd-order ΔΣ ADC is designed in the LP 65 nm CMOS process and occupies an area of 0.221 mm^2^. All circuits shown in Figure 21 are implemented on-chip. A sampling clock is provided from an external clock generator, and all of the other clock sources needed are generated by an on-chip clock generator. All analog and digital circuits in the proposed ADC operate at the 1.2 V supply voltage.

Figure 23b shows the power distribution at the 1.2V supply voltage. The proposed second-order ΔΣ ADC consumes a total power of 530.76 μW at the 1.2 V supply voltage. The chopping CFIA and lossy integrator consume 312 μW and 191.7 μW, which account for 58.8% and 36.1% of total power consumption, respectively. The power consumptions of the chopping CFIA and lossy integrator include bias circuits and common-mode feedback circuits. The SC integrator and the other blocks’ power consumptions are 9.2 μW and 17.9 μW, respectively. The other blocks contain the clock generator, linear VCO-FDSM, and CDAC with reference voltages.

**Figure 24:**
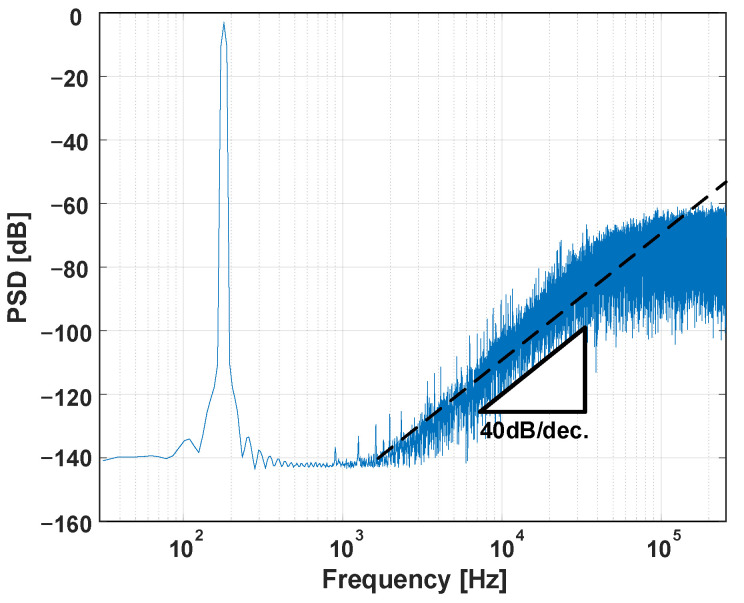
Simulated 2^16^-point 16-averaged spectrum of proposed 2nd-order ΔΣ ADC.

To verify the ADC performance, transient simulation was performed, and the results were converted to a spectrum in the frequency domain through FFT. The transient simulation was performed as the pre-layout circuit and included device noise generated from transistors and resistors. Figure 24 plots the proposed second-order ΔΣ ADC’s 16-average FFT power spectral density (PSD), which comes from 16 different sections in one transient simulation result. The input frequency is 200 Hz, and the input amplitude is −3 dBFS at 1.2 V supply voltage, where the maximum input amplitude is 1.2 V_PP_. The proposed ADC achieves the peak SNDR of 117.7 dB. The spectrum has a 40 dB/decade slope, which is a 2nd-order noise-shaping characteristic. The noise power spectral density normalized to the maximum signal power appears as a noise floor of −143 dB in the frequency domain for a 2^16^-point FFT transformation. However, the designed ADC has a noise floor of approximately −140 dB, as shown in Figure 24. This means that the noise floor of the designed ADC differs by less than 3 dB from the target noise floor.

Table 4 is a performance comparison table of the proposed second-order ΔΣ ADC with other relevant works. The proposed ADC operates at a 1.2 V supply voltage and achieves a peak SNDR of 117.7 dB and a FoM of 180.4 dB, with an OSR of just 256. The proposed ADC architecture demonstrates its advantages in two aspects compared to comparable ADCs. First, ADCs with a low OSR of 256 to 321, as referenced in [30,31,32,33], generally achieve an SNDR limited to around 100 dB. However, the proposed ADC, with an OSR of 256, achieved an SNDR at least 14.7 dB higher than those of the referenced ADCs, with a similar OSR. Moreover, the proposed ADC attains an FoMs of 180 dB, the highest value among ADCs, with an OSR of 256. Second, ADCs targeting an SNDR of 120 dB, as referenced in [21,22], require an OSR of 1000 to 2000. In contrast, the proposed ADC architecture achieves a high SNDR of 117.7 dB with an OSR that is more than four times lower. Additionally, the proposed ADC has an area of 0.221 mm^2^, which is not only the smallest compared to [21,22] but also achieves a high SNDR of 117 dB at a low supply voltage of 1.2 V, making it highly suitable for low-voltage, high-resolution ADC designs. These are the state-of-the-art results among related works.

## 5. Conclusions

A high-resolution second-order ΔΣ ADC has been realized in a 65 nm CMOS technology. The prototype ADC operates at a supply voltage of 1.2 V with a bandwidth of 1 kHz. The target resolution of the proposed ADC is 20 bit. The sampling frequency is 512 kHz, and the OSR is only 256 for a target resolution. This performance is achieved by the proposed filtered gain-stage, which consists of the chopping CFIA, the active lossy integrator, and the linear VCO-FDSM as a multi-bit quantizer. This architecture suffers less from kT/C noise caused by the SC integrator, since the proposed filtered gain stage reduces the input-referred noise of the kT/C noise. The proposed ΔΣ ADC structure provides the possibility of high-resolution ADC design in an environment where the use of a high OSR is limited [40]. The addition of a gain stage requires an additional area for the two OTAs, the resistors, and capacitors for CFIA and LPF configurations. However, to achieve the same performance as a conventional DSM ADC with an OSR of 256, a capacitance of 375 pF is required. Implementing this capacitance still demands a much larger area than the proposed ADC. Therefore, the proposed ADC offers an attractive alternative for achieving high resolution at low OSR.

## Figures and Tables

**Table 1:** N-bit quantizer vs. V_LSB_.

Number of Bits	V_LSB_ (mV)
2	400
3	172
4	180
5	39

**Table 2:** Coefficients of Figure 8a.

Parameter	Value	Equation
f_C_	5 kHz	--
G	50	$G\times a_{1}\times a_{2}=1$
a_1_	0.1	
a_2_	0.2	
K_1_	0.326	$F_{S}/(2\pi \times G\cdot f_{C})$

**Table 3:** Design parameters and noise contribution.

			Simulation	
Block	Parameter	Value	Input-Referred Noise	Noise Contribution
CFIA	$g_{m1}\;\mathrm{of}\;\;\bar{V_{\mathrm{ni},\mathrm{CFIA}}^{2}}$	300 μS	1.542 × 10^−16^	35.2%
	C_IN_	2.5 pF		
	C_F_	0.5 pF		
Active Lossy Integrator	$R_{\mathrm{IN}}\;\mathrm{of}\;\bar{V_{\mathrm{ni},\mathrm{Rin}}^{2}}$	100 KΩ	1.784 × 10^−16^	40.74%
	$g_{m3}\;\mathrm{of}\;\;\bar{V_{\mathrm{ni},\mathrm{Filter}}^{2}}$	30 μS		
	C_Filter_	32 pF		
	R_Filter_	1 MΩ		
SC Integrator	C_S_	300 fF	4.31 × 10^−17^ V^2^/Hz	10%
Quantizer	ENOB_Nyq_	5		>5%
	OSR	256		
Total				90%

**Table 4:** Performance summary and comparison.

	This Work	ISSCC 2006 [30]	JSSC 2017 [31]	VLSI 2018 [32]	VLSI 2016 [33]	JSSC 2018 [22]	JSSC 2013 [21]
Area (mm^2^)	**0.221**	0.06	0.16	0.134	0.5	0.25	0.375
Tech. (nm)	**65**	180	160	65	180	160	160
Architecture	**∆Σ**	∆Σ	Zoom	∆Σ	IADC1 +Multi-Slope	Zoom	Incremental
Supply (V)	**1.2**	0.9	1.8	1.2	1.5	1.8	1.8
BW (kHz)	**1**	10	20	20	1	1	0.000013
Power (μW)	**531**	200	1120	550	34.6	280	6.3
OSR	**256**	256	282	256	321	1000	2000
SNDR (dB)	**117.7**	80.1	103	100.8	96.8	118.1	-
DR (dB)	**-**	83	109	101.8	99.7	120.3	119.8
FoMs (dB)	180.4 **	160 *	181.5 *	177.4 *	174.6 *	185.8 *	182.7 *

${FoM}_{S}^{*}=DR+10log(BW/Power)$ ${FoM}_{S}^{**}=SNDR+10log(BW/Power)$.

## Data Availability

Data are contained within the article.
